# Contrastive Learning with Global and Local Representation for Mixed-Type Wafer Defect Recognition

**DOI:** 10.3390/s25041272

**Published:** 2025-02-19

**Authors:** Shantong Yin, Yangkun Zhang, Rui Wang

**Affiliations:** School of Mechanical Engineering and Automation, Harbin Institute of Technology, Shenzhen 518055, China; styin0425@gmail.com (S.Y.); zhangyangkun@hit.edu.cn (Y.Z.)

**Keywords:** pattern classification and segmentation, contrastive learning, wafer bin map

## Abstract

Recognizing defect patterns in semiconductor wafer bin maps (WBMs) poses a critical challenge in the integrated circuit (IC) manufacturing industry. The accurate classification and segmentation of these defect patterns are of utmost significance as they are key to tracing the root causes of defects, thereby reducing costs and enhancing both product efficiency and quality. As the manufacturing process grows in complexity, the WBM becomes intricate when multiple defect patterns coexist on a single wafer, making the recognition task increasingly complicated. In addition, traditional supervised learning methods require a large number of labeled samples, which is labor-intensive. In this paper, we present a self-supervised contrastive learning framework for the classification and segmentation of mixed-type WBM defect patterns. Our model incorporates a global module for contrastive learning that captures image-level representations, alongside a local module that targets the comprehension of regional details, which is helpful for the segmentation of defective patterns. Experimental results demonstrate that our model performs effectively in scenarios where there is a limited number of labeled examples and a wealth of unlabeled ones.

## 1. Introduction

The production of semiconductor devices is a complex and highly detailed operation, involving hundreds of steps to fabricate integrated circuit (IC) chips on silicon wafers. After wafer fabrication, the wafer needs to be inspected. Common inspection methods include a probing test, scanning electron microscopy (SEM), transmission electron microscopy (TEM), X-ray inspection, and automatic optical inspection (AOI), each generating information at different levels. For probing tests, the electrical performance of each die is tested using wafer probes. Dies that meet quality requirements are cut off from the wafer and utilized as final qualified chips. As the worldwide need for these devices grows, manufacturers are concentrating their efforts on boosting manufacturing efficiency by employing techniques such as maximizing the number of chips per wafer. Nevertheless, the enhanced complexity of these advanced techniques also gives rise to various defects, which are inevitable during the manufacturing processes. Therefore, the results of wafer test are stored in wafer bin maps (WBMs) for defect analysis, contributing to reducing defect rates and ensuring market competitiveness [1].

**Figure 1:**
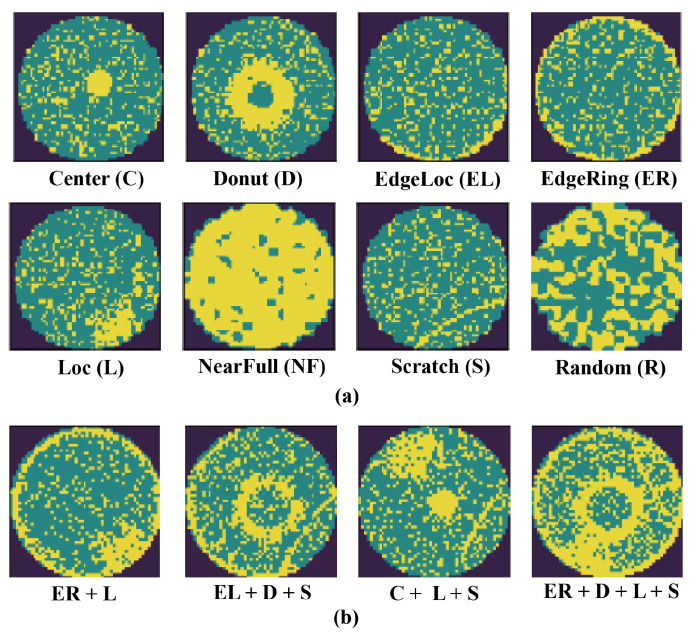
(**a**) Eight common single-type WBM defect patterns. (**b**) Examples of mixed-type WBM defect patterns.

WBM defect pattern recognition is crucial for detecting systemic manufacturing issues, as the accurate classification and segmentation results of WBM defect patterns can effectively infer the root causes of defects in the manufacturing process [2]. The defects on the wafer can generally be divided into two categories: random errors and systematic faults. When dealing with wafer defect patterns, the analysis of systematic faults is generally the main focus, as the defect patterns caused by random errors are irregular and difficult to eliminate. As shown in Figure 1a, the typical single defect patterns contain the following: “Center”, “Donut”, “EdgeLoc”, “EdgeRing”, “Loc”, “NearFull”, “Scratch”, and “Random” [3]. With the ongoing advancement in the IC industry, the complexity of manufacturing raises the likelihood of mixed-type WBM defect patterns appearing on the same wafer [4,5], as shown in Figure 1b. These defect patterns may interact with each other, resulting in more intricate issues. Consequently, accurate classification and segmentation of these mixed-type WBM defect patterns are essential for effective quality control.

Given the intricate nature of mixed-type WBM defect patterns, manual inspection becomes increasingly labor-intensive and time-consuming [6]. Alternative solutions are required in industrial production. Recent studies have explored the application of artificial intelligence and computer vision models for defect pattern analysis [2]. However, existing research mainly focuses on image classification and cannot accurately identify the size and location of mixed-type defect patterns. In addition, training a model to recognize defect patterns often requires a large number of labeled samples and undoubtedly wastes manpower and resources. Self-supervised learning can learn from unlabeled data, which is particularly attractive in situations where it is difficult to obtain large amounts of labeled data [7]. Although self-supervised learning is currently mainly used for image classification tasks, our work is based on the intuition that the WBM defect pattern segmentation can also benefit from representations learned through self-supervised learning from unlabeled data.

This study centers on contrastive learning, a branch of self-supervised learning [8,9]. According to our research, most existing contrastive learning approaches prioritize global representations, which are not optimal for segmentation tasks that require pixel-level distinctions [10]. If local regions of images are extracted for contrastive learning, then local representations can be adequately learned [11] for segmentation tasks. Consequently, we propose a self-supervised contrastive learning model that integrates both global and local contrastive learning modules, with queue mechanisms introduced to decouple the sample size from mini-batch constraints. Specifically, the global contrastive learning module focuses on the learning of global representations, while the local contrastive learning module is used to learn pixel-level discrimination. With the introduction of queues, each module receives a sufficient number of negative samples, improving the contrastive loss computation. To the best of our knowledge, this is the first application of contrastive learning to both the classification and segmentation of WBM defect patterns. The primary contributions of this paper are as follows:

(1) We employ self-supervised contrastive learning for both the classification and segmentation of mixed-type defect patterns. In this way, we can obtain more precise information about the types and locations of different defect patterns.
(2) Our proposed model integrates global and local contrastive learning modules, enabling the learning of both image-level and pixel-level features directly from unlabeled data. This approach provides a robust foundation for downstream classification and segmentation tasks using only a limited amount of labeled data.
(3) Experimental evaluations on the mixed-type WBM defect pattern dataset demonstrate that our framework outperforms existing self-supervised methods.

The remainder of this paper is organized as follows: Section 2 presents a brief overview of recent studies on mixed-type WBM defect pattern classification and segmentation. Section 3 details the proposed methodology. Section 4 discusses the experimental results and analysis. Finally, Section 5 contains concluding remarks and directions for future research.

## 2. Related Works

### 2.1. WBM Defect Pattern Recognition

WBM defect pattern analysis is crucial for identifying the root cause of process failures in semiconductor manufacturing. The current research mainly focuses on the classification of single defect patterns and can be roughly divided into three categories. The first category mainly monitors defect patterns on WBMs by constructing statistical information [12,13], which can successfully separate normal and abnormal graphic patterns, but it is difficult to distinguish different defect patterns. The second category is model-based clustering methods. For example, Wang et al. [14] used Gaussian EM to detect elliptical and linear patterns and used the shell algorithm to estimate annular patterns. Yuan and Kuo [15] proposed a mixed model describing the distribution of defects and used Bayesian inference to estimate parameters. The last category is machine learning methods that have received more and more attention in recent years. Wang and Chen [16] trained a multilayer perceptron (MLP) on a set of hand-crafted spatial filters that reflect the rotational invariance property of WBM. Kulkarni [3] applied CNNs to classify defect patterns based on a synthesized WBM dataset. Kang et al. [17] presented a semi-supervised representation learning method, which fully utilizes the information in both unlabeled and labeled wafer maps. Most of the above research relies on large WBM databases and has problems with unsatisfactory performance and high computational costs.

The composition of mixed-type WBM defect patterns is more complex, as the combination of defect patterns can vary greatly. Thus, research on the classification of mixed-type WBM defect patterns is less extensive than that on single WBM defect patterns. In these studies, Byun and Baek [18] proposed a new approach using a convolutional autoencoder to initialize the weights of a CNN. Tello et al. [19] used feature rules to determine whether a wafer map has mixed-type patterns and then used a stochastic general regression neural network (GRNN) and a deep structured CNN for classification. Kyeong and Kim [20] applied a CNN to classify WBM defects with mixed-type defect patterns. Wang et al. [5] proposed DC-Net, which used a deformable convolutional network (DC-Net) to classify mixed-type defect patterns. These methods are unable to recognize and locate mixed-type defect patterns on WBMs simultaneously, and the research on the separation of multiple defect patterns is still insufficient.

Compared with classification, image segmentation can provide more detailed information and better understand the content of the image. For mixed-type WBM defect pattern segmentation, Nag et al. [21] recently proposed a novel network based on an encoder–decoder architecture for the simultaneous classification and segmentation of single and mixed defect patterns. Chiu et al. [2] proposed a method based on Mask R-CNN [22] to identify WBM defect patterns. Yan et al. [23] presented a new U-Net framework that used semantic segmentation methods to segment different defect patterns on WBMs. However, existing research tends to focus more on global image representation while overlooking the powerful auxiliary role that local information plays in image segmentation tasks.

### 2.2. Self-Supervised Contrastive Learning

**Figure 2:**
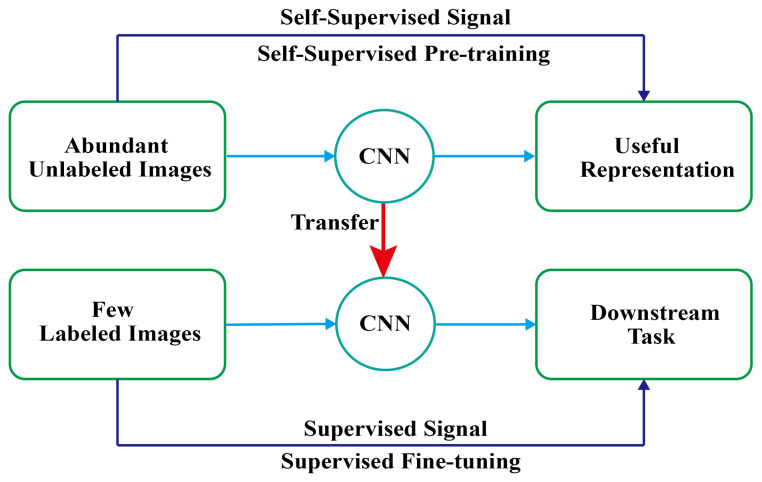
The self-supervised learning paradigm.

Self-supervised learning mainly uses auxiliary tasks to mine its own supervision information from large-scale unsupervised data and trains the network through this constructed supervision information [7,24]. As shown in Figure 2, the core idea of the self-supervised learning paradigm is to first learn knowledge from unlabeled samples by designing self-supervised signals and then transfer it to downstream tasks. As a branch of self-supervised learning, contrastive learning [25] focuses on learning the common features between instances of the same class and distinguishing the differences between instances of different classes. Contrastive learning can effectively utilize unlabeled data for training [9,26] and has been widely applied in the field of natural image processing. In the field of WBM defect pattern classification, Kwak et al. [27] applied a method (SWaCo) for safe WBM classification with self-supervised contrastive learning to effectively exploit unlabeled data. Kahng et al. [28] presented a self-supervised learning framework that fully utilizes unlabeled data to pre-learn rich visual representations for data-efficient WBM defect pattern classification. Hu et al. [29] proposed a contrastive learning framework for semi-supervised learning and prediction of WBM defect patterns. It can be seen that self-supervised contrastive learning methods do not heavily rely on the number of labeled samples and are mostly used for the classification of WBM defect patterns, but there is still little research on segmentation tasks. The urgent challenge lies in establishing a self-supervised contrastive learning model that possesses both classification and segmentation capabilities for mixed-type WBM defect patterns.

## 3. Methodology

### 3.1. Overview

**Figure 3:**
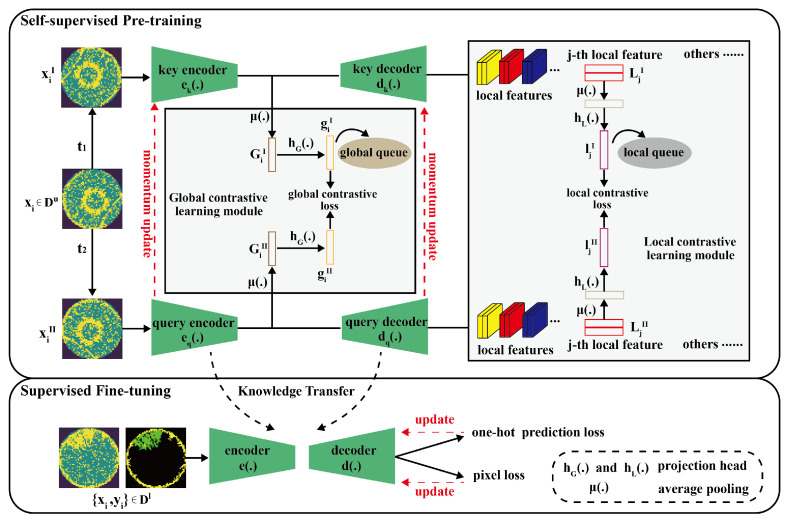
The overall framework of our proposed model. Note that the figure omits the display of negative samples. In the self-supervised pre-training stage, we pre-train the network using a large amount of unlabeled data $x_{i}\in D^{u}$. After that, we transfer the knowledge embedded in the query encoder and query decoder to the downstream task and fine-tune the network with few labeled samples $\left\{x_{i},y_{i}\right\}\in D^{l}$.

In this paper, we propose a classification and segmentation model for mixed-type WBM defect patterns based on self-supervised contrastive learning. In our problem setting, we consider a set of WBM data including *N* labeled data and *M* unlabeled data, where N≪M. For simplicity, the unlabeled set is defined as $D^{u}={\left\{x_{i}\right\}}_{i=1}^{M}$, and the labeled set is defined as $D^{l}={\left\{x_{i},y_{i}\right\}}_{i=M+1}^{M+N}$. As shown in Figure 3, the framework consists of a self-supervised pre-training stage and a supervised fine-tuning stage. In pre-training, global contrastive learning module is designed to learn image-level representation, while the local contrastive learning module is used to better understand the structure of local regions, which contributes to the image segmentation task. To distinguish extracted features with different connotations, we apply the concept of a query and a key to describe encoders and decoders, although they have the same structure respectively. A query can be understood as the target to be searched for, and a key is the template. The model is built by matching the query to a dictionary of keys, which is a queue of the encoded representations of data samples that is updated with each mini-batch. In fine-tuning, pre-trained weights are transferred to the downstream classification and segmentation tasks and fine-tuned with a limited number of labeled samples. This section describes the proposed framework in detail.

### 3.2. Data Augmentation

**Figure 4:**
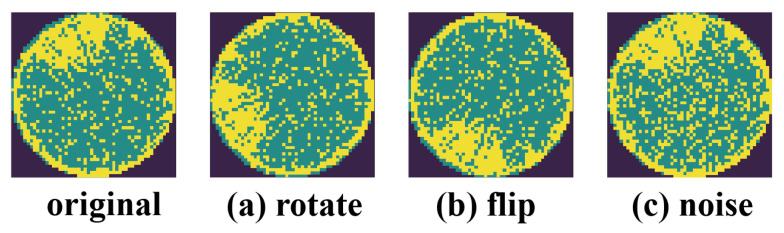
Illustration of the data augmentation operators.

In contrastive learning, the model is trained by enforcing similarity between positive sample pairs and dissimilarity between negative sample pairs. Therefore, the core of contrastive learning is the construction of positive and negative samples. In practice, different augmented views of the same sample after data augmentation are seen as positive samples, while other samples are defined as negative samples. Supported by data augmentation, the model can learn better feature representations of the data, capture the fundamental structure and relationships between samples, and effectively prevent over-fitting. As shown in Figure 4, we perform data augmentation operators, i.e., random rotation, flipping, and random noise on samples for learning the rotational invariance of WBMs and improving the robustness and generalization ability of the model. Regarding the noise addition operations in data augmentation, given that noise in semiconductor manufacturing environments (such as dust, light variations, etc.) can affect the performance of detection systems, we introduce different types of artificial noise (Gaussian Noise, Salt-and-Pepper Noise, and Poisson Noise) to simulate real-world production noise conditions. Specifically, given unlabeled sample $x_{i}\in D^{u}$, two augmented WBMs $x_{i}^{I}$ and $x_{i}^{\mathrm{II}}$ are generated by data augmentation $t_{1}$ and $t_{2}$, i.e., $x_{i}^{I}=t_{1}\left(x_{i}\right)$ and $x_{i}^{\mathrm{II}}=t_{2}\left(x_{i}\right)$. These two augmented views are then used for the illustration of subsequent feature extraction.

### 3.3. Global Contrastive Learning Module

The global contrastive learning module mainly promotes the model to learn the ability of extracting global features. Since global features provide the context information of the image, the designing of this module contributes to the effective comprehension of the overall structure of mixed-type WBMs. Specifically, key and query encoders are used to extract high-dimensional key and query global features. Then, these high-dimensional features are mapped to lower-dimensional hidden spaces for a more compact representation through the global projection head. After that, the global queue that is a unique form of a linear list is employed to dynamically manage key features. The construction of the global contrastive learning module is similar to MoCo v2 [8]; we provide a detailed description as follows.

#### 3.3.1. Global Feature Extraction

Global features refer to the overall attributes of an image, capable of capturing the global information and understanding the image from a holistic perspective. We apply the concept of a query and a key to describe encoders for distinguishing extracted features with different connotations. As given a series of key features and a query feature, contrastive learning is to find the unique key feature that matches the query feature the most and enforce them to be similar while distancing from others.

Firstly, a key encoder $e_{k}\left(\cdot \right)$ and a query encoder $e_{q}\left(\cdot \right)$ are used to obtain global key feature $G_{i}^{I}$ and global query feature $G_{i}^{\mathrm{II}}$ from the augmented sample instances, respectively. The procedure can be formulated as

(1) $$G_{i}^{I}=\mu \left(e_{k}\left(x_{i}^{I}\right)\right),G_{i}^{\mathrm{II}}=\mu \left(e_{q}\left(x_{i}^{\mathrm{II}}\right)\right),$$

where μ(·) represents the average value of each channel in the feature map, i.e., the global average pooling. $e_{k}\left(\cdot \right)$ denotes the key encoder and $e_{q}\left(\cdot \right)$ denotes the query encoder, which have the same structure as the downstream encoder e(·).

In addition, the global projection head $h_{G}\left(\cdot \right)$ is an MLP with one hidden layer (with ReLU) in this module, whose presence has been proven to be beneficial to push the dissimilar samples away from each other and obtain more useful information for downstream tasks [9]. The global projection head can map the high-dimensional features $G_{i}^{I}$ and $G_{i}^{\mathrm{II}}$ to lower-dimensional hidden spaces for a more compact representation, encouraging our model to learn more abstract and informative features. Therefore, we obtain the final global key feature $g_{i}^{I}$ and global query feature $g_{i}^{\mathrm{II}}$ by passing $G_{i}^{I}$ and $G_{i}^{\mathrm{II}}$ through the global projection head $h_{G}\left(\cdot \right)$ to further calculate the global contrastive loss. The conversion can be formulated as follows:

(2) $$g_{i}^{I}=h_{G}\left(G_{i}^{I}\right)=W_{G}^{\left(2\right)}R_{G}\left(W_{G}^{\left(1\right)}G_{i}^{I}\right),$$

(3) $$g_{i}^{\mathrm{II}}=h_{G}\left(G_{i}^{\mathrm{II}}\right)=W_{G}^{\left(2\right)}R_{G}\left(W_{G}^{\left(1\right)}G_{i}^{\mathrm{II}}\right),$$

where $W_{G}^{\left(1\right)}$ and $W_{G}^{\left(2\right)}$ are linear transformation matrices, and $R_{G}$ is a ReLU nonlinearity.

Furthermore, the global queue that is a unique form of a linear list for dynamically managing vectors is introduced, as shown in Figure 3. Since the core of contrastive learning is to construct sufficient negative sample pairs, we design the global queue to store abundant negative vectors (i.e., key features) generated by the key encoder. The size of key features in the global queue can far exceed the capacity of a single batch, which can increase the diversity of training and improve the generalization ability of the model. Therefore, the introduction of the queue can decouple the negative vector size from the mini-batch size. In the dynamic management of the global queue, the current mini-batch of encoded key features is enqueued into the queue and the oldest key features are dequeued from the queue. This process ensures that the model is always learning from the latest data by a dynamic and efficient management of key features.

#### 3.3.2. Global Contrastive Loss

Global contrastive loss is a measure of the similarities between global features in a representation space and promotes the model to learn global representations. In contrastive learning, if a query feature (generated by query encoder) is given, only a unique key feature in the global queue can match it. So, the idea of contrastive learning is to compare the query feature with all the key features in the queue and hope that the query feature is as similar as possible to its matching key feature, and then it stays away from others. Consider an encoded query feature $g_{i}^{\mathrm{II}}$ and a set of encoded samples $\left\{g_{0}^{I},\ldots \;g_{i}^{I},\ldots \;g_{K}^{I}\right\}$ that contains K+1 key features in the global queue. Obviously, there is a single key feature (denoted as $g_{i}^{I}$) that matches $g_{i}^{\mathrm{II}}$. Then, the contrastive loss function, called InfoNCE [30], is used to expect matching pairs to be similar and mismatching pairs to be dissimilar, as follows:

(4) $$L_{G}=\frac{1}{N_{G}}\sum_{i=1}^{N_{G}}-\log\frac{\exp\left(g_{i}^{\mathrm{II}}\cdot g_{i}^{I}/\tau_{g}\right)}{\sum_{k=0}^{K}\exp\left(g_{i}^{\mathrm{II}}\cdot g_{k}^{I}/\tau_{g}\right)},$$

where $\tau_{g}$ is a temperature hyper-parameter, and $N_{G}$ denotes the number of samples from a mini-batch. The sum in the denominator is calculated over one positive key feature $g_{i}^{I}$ and other *K* negative key features. Intuitively, the InfoNCE loss tries to classify the query feature $g_{i}^{\mathrm{II}}$ as its corresponding positive key feature $g_{i}^{I}$ rather than others.

### 3.4. Local Contrastive Learning Module

The local contrastive learning module is a local contrastive representation space for computing the contrastive loss associated with the local regions of a WBM. For a WBM with mixed-type defect patterns, the segmentation task is more complex due to the interconnected nature of defects and their variability in quantity, position, and type, etc. While global features can effectively capture the overall information of an image, relying solely on global features to measure WBM defect patterns will result in the neglect of crucial local details. This approach may not be optimal for segmentation tasks that require pixel-level discrimination. To address this issue, we design the local contrastive learning module to learn the representation of local regions. We separate the WBM into multiple regions, selectively identify relevant regions, and subsequently perform position matching and feature extraction for local contrastive learning. This method ensures that the segmentation process benefits from both the global context and the fine-grained local information, enhancing the overall accuracy and effectiveness of defect recognition. The details are as follows:

#### 3.4.1. Local Region Selection and Matching

**Figure 5:**
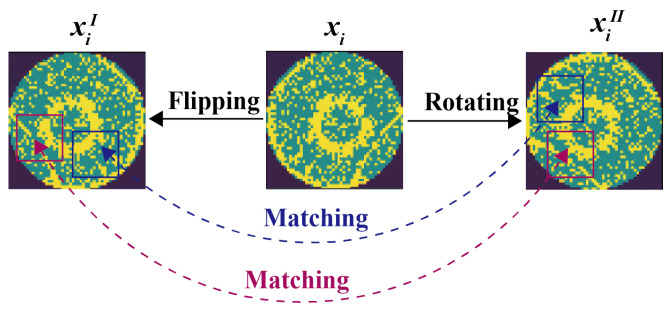
Process of local region selection and matching.

Randomly selected local regions will be biased towards the more dominant feature categories, as the “background” category accounts for a large proportion in mixed-type WBM defect patterns. Therefore, we need to make the selection of local regions reasonable to contain more valuable feature categories. For example, as shown in Figure 5, flipping and rotating operations are performed on $x_{i}$, and two augmented views $x_{i}^{I}$ and $x_{i}^{\mathrm{II}}$ are obtained respectively. The selected local regions are then matched according to their positions.

The local region selection and matching is performed by the selection of appropriate regions in $x_{i}^{I}$ at first, and then by the determination of the local regions in $x_{i}^{\mathrm{II}}$ that match with the selected regions. The appropriateness of each local region to be selected is evaluated by its weight, which is calculated by a convolutional layer with the kernel size being the same as the local region size $n_{s}\times n_{s}$. Specifically, $x_{i}^{I}$ is input into the convolutional layer, and the weight of each local region is calculated and recorded by sliding the kernel. We select the local region with the maximum weight from $x_{i}^{I}$, and then the position of the matching local region in $x_{i}^{\mathrm{II}}$ is determined. To minimize overlap, after each local region selection, the region is excluded so that the centers of subsequently selected regions do not fall into the previously selected local regions. This weighted selection and matching process is repeated $n_{r}$ times, selecting distinct local regions in each iteration to obtain $n_{r}$ pairs of matched local regions from images $x_{i}^{I}$ and $x_{i}^{\mathrm{II}}$.

#### 3.4.2. Local Feature Extraction

Local features $l_{j}^{I}$ and $l_{j}^{\mathrm{II}}$ that capture the pixel-level discrimination of WBM defect patterns are extracted by the following steps. Firstly, feature maps $d_{k}\left(e_{k}\left(x_{i}^{I}\right)\right)$ and $d_{q}\left(e_{q}\left(x_{i}^{\mathrm{II}}\right)\right)$ of a positive pair ($x_{i}^{I}$, $x_{i}^{\mathrm{II}}$) are obtained from a key and query encoder–decoder network. Here, $e_{k}\left(\cdot \right)$ and $e_{q}\left(\cdot \right)$ are key and query encoders while $d_{k}\left(\cdot \right)$ and $d_{q}\left(\cdot \right)$ are key and query decoders, which have the same structure as the downstream encoder–decoder network, respectively. Then, the *j*-th $\left(j=1,\ldots ,n_{r}\right)$ pair of matching local region feature maps $L_{j}^{I}$ and $L_{j}^{\mathrm{II}}$ is extracted according to the idea of local region selection and matching. After that, average pooling μ(·) is performed on local region feature maps $L_{j}^{I}$ and $L_{j}^{\mathrm{II}}$, and the results then pass through the local projection head $h_{L}\left(\cdot \right)$. Therefore, the final local features can be defined as follows:

(5) $$l_{j}^{I}=h_{L}\left(\mu \left(L_{j}^{I}\right)\right)=W_{L}^{\left(2\right)}R_{L}\left(W_{L}^{\left(1\right)}\mu \left(L_{j}^{I}\right)\right),$$

(6) $$l_{j}^{\mathrm{II}}=h_{L}\left(\mu \left(L_{j}^{\mathrm{II}}\right)\right)=W_{L}^{\left(2\right)}R_{L}\left(W_{L}^{\left(1\right)}\mu \left(L_{j}^{\mathrm{II}}\right)\right),$$

where feature maps $d_{k}\left(e_{k}\left(x_{i}^{I}\right)\right)$ and $d_{q}\left(e_{q}\left(x_{i}^{\mathrm{II}}\right)\right)$ have the same size as the original image. The local projection head $h_{L}\left(\cdot \right)$ can contribute to making the model better capture the differences between local regions. μ represents calculating the average value of each channel in feature maps, $W_{L}^{\left(1\right)}$ and $W_{L}^{\left(2\right)}$ are linear transformation matrices, and $R_{L}$ is a ReLU nonlinearity. Similar to the global contrastive learning, the queue is also applied to dynamically store and manage local region vectors in the training of the model, as shown in Figure 3.

#### 3.4.3. Local Contrastive Loss

The local contrastive loss updates the network by forcing the feature representations of matching local regions to be similar and the feature representations of different local regions to be dissimilar. Specifically, considering a local query feature $l_{j}^{\mathrm{II}}$ and a set of vectors $\left\{l_{0}^{I},\ldots \;l_{j}^{I},\ldots \;l_{\Gamma }^{I}\right\}$ that contain Γ+1 key features in the local queue, there is a single key feature (denoted as $l_{j}^{I}$) that matches $l_{j}^{\mathrm{II}}$. Then, the local contrastive loss can also be calculated by InfoNCE as follows:

(7) $$L_{L}=\frac{1}{N_{L}}\sum_{j=1}^{N_{L}}-\log\frac{\exp\left(l_{j}^{\mathrm{II}}\cdot l_{j}^{I}/\tau_{l}\right)}{\sum_{\gamma =0}^{\Gamma }\exp\left(l_{j}^{\mathrm{II}}\cdot l_{\gamma }^{I}/\tau_{l}\right)},$$

where $\tau_{l}$ is a temperature hyper-parameter, $N_{L}$ indicates the number of pairs of matched local regions in a mini-batch, and Γ represents the number of key features in the local queue.

### 3.5. Network Updating

#### 3.5.1. Self-Supervised Pre-Training

In the pre-training period, the total contrastive loss function to update the query encoder parameters $\theta_{e_{q}}$ and query decoder parameters $\theta_{d_{q}}$ is defined as follows:

(8) $$L_{P}=\lambda_{1}L_{G}+\left(1-\lambda_{1}\right)L_{L},$$

where $L_{G}$ represents the global contrastive loss in Equation (4), and $L_{L}$ represents the local contrastive loss in Equation (7).

Although using queues allows for the size of negative vectors to be larger, it also poses difficulties for updating the key encoder and key decoder via back-propagation, since the gradient should propagate to all samples in the queue. To address this issue, a momentum update approach is utilized for the key encoder and decoder, which accumulates exponential moving averages of the key network to ensure a stable and efficient update manner. To be specific, the query encoder parameters $\theta_{e_{q}}$ and the decoder parameters $\theta_{d_{q}}$ are updated via back-propagation by Equation (8), while the key encoder parameters $\theta_{e_{k}}$ and decoder parameters $\theta_{d_{k}}$ are updated by the momentum equations:

(9) $$\theta_{e_{k}}\leftarrow \alpha_{1}\theta_{e_{k}}+\left(1-\alpha_{1}\right)\theta_{e_{q}},$$

and

(10) $$\theta_{d_{k}}\leftarrow \alpha_{2}\theta_{d_{k}}+\left(1-\alpha_{2}\right)\theta_{d_{q}},$$

where $\alpha_{1}$ and $\alpha_{2}$∈[0,1) are the momentum coefficients. The momentum updating makes parameters $\theta_{e_{k}}$ and $\theta_{d_{k}}$ evolve more smoothly than $\theta_{e_{q}}$ and $\theta_{d_{q}}$. Therefore, although the keys in the queue are generated by different encoders and decoders, the differences between these encoders and decoders can be ignored.

#### 3.5.2. Supervised Fine-Tuning

Supervised fine-tuning refers to training the model on a smaller and task-specific labeled dataset. The purpose of this stage is to make the model adapt to the specific requirements of the given task. In detail, the model retains the knowledge learned in the pre-training stage while also learning additional information relevant to the specific task. In our problem, given labeled data $\left\{x_{i},y_{i}\right\}\in D^{l}$, we fine-tune the whole network through a segmentation loss $L_{S}$ and classification loss $L_{C}$.

For the computation of the segmentation loss $L_{S}$, we employ the cross-entropy loss function [31], which computes the pixel-level difference between segmented defect patterns and the ground truth. However, merely calculating the loss value of pixels may result in over-segmentation. It is still necessary to compute the loss value associated with the type of defect. We use a *C*-bit one-hot value to represent the predicted types of WBM defect patterns and calculate the corresponding binary cross entropy loss $L_{C}$. Since we need to simultaneously focus on the classification and segmentation of WBM defect patterns, the total loss function in the fine-tuning period is defined as follows:

(11) $$L_{F}=\lambda_{2}L_{S}+\left(1-\lambda_{2}\right)L_{C},$$

where $L_{S}$ represents the segmentation loss and $L_{C}$ represents the classification loss.

## 4. Experimental Results

### 4.1. Data Description

**Figure 6:**
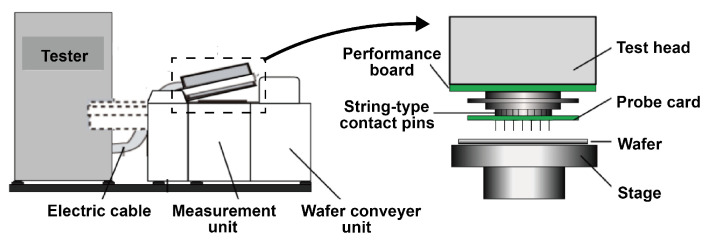
Wafer probe testing station.

A wafer bin map (WBM) is the result of the circuit probing process, recording the spatial distribution of defective chips on the wafer. The WBMs dataset collection system is shown in Figure 6. The primary method of wafer testing involves the coordination between the tester and the probe stage [32]. During the testing process, the tester cannot directly measure the wafer under test. Instead, it relies on the probes in the probe card to make electrical contact with the pads or bumps on the wafer. The test signals measured by the probes are then sent to the automatic test equipment (ATE) for analysis and judgment, thereby obtaining the electrical characteristics test results for each die on the wafer. All samples are collected in actual industrial scenarios by the wafer probe testing station. We standardized the image size to 52×52.

As shown in Table 1, the defect types contain 8 kinds of single defect patterns and 29 kinds of mixed-type defect patterns (13 kinds of double mixed-type defect patterns, 12 kinds of triple mixed-type defect patterns, and 4 kinds of quadruple mixed-type defect patterns). We represent each type of defect using the uppercase initial letter of its name as shown in Figure 1. It is worth noting that Random and NearFull defects do not significantly point to specific manufacturing roots, and their impact in practice is minimal. Also, these two types of patterns are not suitable for generating mixed defects with other single defect patterns, so we do not consider the synthesis of these two types. Furthermore, we consider the WBMs of the normal category (C0) into the model training process, and there are 37 kinds of defect patterns and 1 kind of normal category in total. In the final dataset, we collected 19,000 WBMs with 500 instances for each defect pattern. In our problem setting, the original WBMs have only pixel values 0, 1, and 2, so $x_{i}\in {\left\{0,1,2\right\}}^{H\times W}$ mentioned earlier is the WBM; $y_{i}=\left\{\left(m_{i},s_{i}\right)|m_{i}\in {\left\{0,1\right\}}^{C\times H\times W},s_{i}\in {\left\{0,1\right\}}^{C}\right\}$ is the corresponding ground truth category label, where *C* denotes the number of pattern types; $m_{i}$ is the pixel label map of the *i*-th sample; and $s_{i}$ represents the *C*-bit one-hot label.

### 4.2. Model Evaluation

In this paper, we focus on both the segmentation and classification performance of mixed-type WBM defect patterns. Specifically, we use Pacc (pixel predicted accuracy) and IoU (Intersection over Union) to evaluate the results of semantic segmentation, which can be defined for a specific class *c* as follows:

(12) $$Pacc_{i}^{c}=\frac{Area({\hat{m}}_{i}^{c}\cap m_{i}^{c})}{H\times W},$$

(13) $$IoU_{i}^{c}=\frac{Area({\hat{m}}_{i}^{c}\cap m_{i}^{c})}{Area({\hat{m}}_{i}^{c}\cup m_{i}^{c})},$$

where $m_{i}^{c}$ and ${\hat{m}}_{i}^{c}$ denote the actual and predicted pixel label maps of the *c*-th pattern in the *i*-th WBM. For mixed-type patterns, the mean IoU (mIoU) and the mean Pacc (mPacc) are used to evaluate the model segmentation performance.

For the evaluation of classification results, we use Accuracy to measure the classification accuracy of mixed-type WBM defect patterns. To evaluate the performance in the false classification of WBMs, Precision and Recall are employed in our experiments. The definitions of the indices are shown as follows:

(14) $$Accuracy=\frac{TP+TN}{TP+TN+FP+FN},$$

(15) $$Precision=\frac{TP}{TP+FP},$$

(16) $$Recall=\frac{TP}{TP+FN}.$$

where TP denotes the true positives, TN is the true negatives, FP represents the false positives, and FN represents false negatives.

### 4.3. Performance and Analysis

#### 4.3.1. Implementation Details

In this work, the encoders and decoders designed in our model have the same structure as those in DeepLab v3+ [33]. During the pre-training period, we used an abundant amount of unlabeled WBMs to pre-train the encoder and decoder for 350 epochs with a batch size of 64. We optimized the parameters with Adam optimizer, and the initial learning rate was set as 0.01 with the cosine decay schedule. In addition, we selected 6 local regions of size 12×12 from each sample in the local region selection and matching process (i.e., $n_{s}=12$, $n_{r}=6$). The hyper-parameter $\lambda_{1}$ in Equation (8) was set to 0.5 to balance the global contrast loss and local contrast loss. Following [8], the momentum coefficients $\alpha_{1}$ and $\alpha_{2}$ were all set to 0.999, and the size of the queues was set to 2048. After pre-training, the model with the lowest loss was saved for the downstream segmentation task.

In the fine-tuning period, we trained our model using a small amount of labeled WBMs for a total of 150 epochs, with a batch size of 32. The initial learning rate was set to 0.001, which decreased to 98% per epoch. The hyper-parameter $\lambda_{2}$ in Equation (11) is used to balance pixel loss and one-hot prediction loss, so we also set it to a default 0.5. In addition, the experiment was implemented on an equipment with an Intel(R) Xeon(R) Gold 6226R @ 2.90 GHz CPU and an NVIDIA RTX A6000 GPU by Python 3.8.3.

#### 4.3.2. Model Performance

In our experimental setting, we randomly split the dataset into three subsets: 80% for pre-training, 10% for fine-tuning, and 10% for testing. We obtained the Pacc, IoU, Accuracy, Precision, and Recall for 38 kinds of defect patterns to demonstrate the performance of our model. Table 2 primarily displays the segmentation and classification results for the normal pattern and the single-type defect patterns, Table 3 shows the segmentation results for the mixed-type defect patterns, and Table 4 presents the classification results for the mixed-type defect patterns. As shown in Table 2, the horizontal axis represents different categories (C0 to C8), while the vertical axis displays various evaluation metrics for segmentation and classification. The average Pacc, IoU, Accuracy, Precision, and Recall of our proposed model for C0–C8 are 95.78%, 93.87%, 96.80%, 94.61%, and 94.89%. In Table 3 and Table 4, to facilitate the presentation of the experimental results, the horizontal axis represents various evaluation metrics for segmentation and classification, respectively, while the vertical axis corresponds to different categories (C9 to C37). The average Pacc, IoU, Accuracy, Precision, and Recall for C9–C37 are 95.40%, 90.51%, 92.34%, 94.45%, and 94.66%. From an overall perspective, the more the defect classes that appear on the same WBM, the more difficult it is to recognize and segment. However, whether it is a mixture of two defects, three defects, or four defects, our model accurately accomplished the recognition and segmentation of WBMs in the testing set.

**Figure 7:**
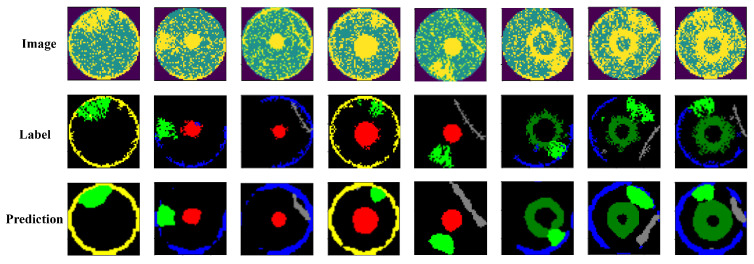
Examples of visualization results obtained using our proposed model.

Figure 7 presents several mixed-type WBM segmentation results achieved with our proposed model. We randomly selected eight experimental samples, each displaying the original mixed-type WBM, label map, and corresponding segmentation output. We can see that the segmentation maps obtained using our model are smooth, complete, and robust to noise. Even in the cases where several mixed defect patterns are overlapped, our model can still distinguish them based on the different characteristics of each defect pattern. For all these defect patterns, the *C* and ER classes are best classified and segmented. This is because the location and shape of the *C* and ER defect patterns are relatively fixed and easier to distinguish. Even for the *S* class, which may easily interact with other pattern classes, our model still has an excellent segmentation performance. Therefore, our model has great potential and application value in the field of WBMs classification and segmentation.

#### 4.3.3. Comparison with Other Methods

In this section, we compare the performance of our proposed model with other self-supervised learning models, including Jigsaw [34], SimCLR [9], and MoCo v2 [8]. These are all relatively successful contrastive learning models. To ensure fairness in comparison, all models used in the experiments have the same dataset and division ratio as in the previous section.

As shown in Table 5, the horizontal axis represents the overall evaluation metrics for segmentation and classification, including mPacc, mIoU (used for segmentation), and Accuracy, Precision, and Recall (used for classification), while the vertical axis displays the numerical results for each method across these metrics. The overall mPacc, mIoU, Accuracy, Precision, and Recall values of our model are as high as 95.59%, 92.19%, 93.57%, 94.43%, and 94.78%, respectively. These indicators are far superior to the other three self-supervised contrastive learning methods. SimCLR performs the worst, with the overall IoU and Accuracy values only being 75.85% and 80.41%. This is related to the fact that SimCLR relies more on large batch data, and the model cannot learn more useful knowledge from limited data. Although self-supervised contrastive learning is widely used in the field of image classification, research on segmentation tasks is still somewhat lacking. The other three common contrastive learning methods used for comparison only focus on global representations; thus, they ignore the local information, which is important for segmentation tasks. So, even after the fine-tuning period, their performance is still not excellent. However, our approach is designed to focus on both the global and local information of the image. Therefore, the proposed model can perform well whether the WBM defect patterns are easy or difficult to distinguish.

### 4.4. Discussion

#### 4.4.1. Effectiveness of the Amount of Self-Supervised Unlabeled Data

Self-supervised pre-training needs to use a large amount of unlabeled data, and an abundance of unlabeled WBMs is readily accessible in the industrial manufacturing process. Therefore, this section investigates the potential of more self-supervised pre-training unlabeled data improving the performance of the model. Building upon our WBM dataset, we randomly utilized 1%, 25%, 50%, 75%, and 100% pre-training unlabeled WBMs. As presented in Table 6, the horizontal axis represents different amounts of pre-training unlabeled data, while the vertical axis displays various evaluation metrics for segmentation and classification. The experimental results indicate an overall upward trend as the amount of unlabeled data increases. This is because more unlabeled pre-training data contain richer features, so the model can learn more distinctive feature representations, thereby improving the generalization ability. Hence, it can be anticipated that the proposed method may yield even greater benefits when applied to a larger dataset for self-supervised pre-training.

#### 4.4.2. Effectiveness of the Amount of Fine-Tuning Labeled Data

This section examines the influence of utilizing additional labeled data for fine-tuning on model performance. Initially, we expanded the fine-tuning labeled dataset to the same size as the pre-training unlabeled dataset. Then, we fine-tuned the model using varying proportions of labeled data, specifically 1%, 5%, 10%, 25%, 50%, and 100% of the self-supervised unlabeled data. The results, presented in Table 7, indicate that as the amount of labeled fine-tuning data increased, the evaluation metrics exhibited an upward trend, although at a diminishing rate. This can be attributed to the model’s ability to learn more information with an increasing number of labels but with a diminishing rate for each additional label. When the amount of labeled data reaches 25% of the self-supervised unlabeled data, the model achieves outstanding performance, and almost all of the evaluation metrics reach a fairly satisfactory result. Therefore, our proposed self-supervised contrastive learning model is not heavily dependent on the size of the labeled data but still remains effective even when a large amount of labeled data are available.

#### 4.4.3. Ablation Study

In this section, we describe ablation experiments we conducted to investigate the effectiveness of the modules in our proposed method. Our proposed model mainly consists of three parts: global contrastive learning module, local contrastive learning module, and fine-tuning module. We chose to remove one or two modules separately for experimental research. The experimental results are shown in Table 8. We present the results of the ablative experiments conducted to assess the effectiveness of different modules. The horizontal axis represents the evaluation metrics, while the vertical axis lists various combinations of the modules. When there is only the global contrastive learning module or only the local contrastive learning module, the performance is slightly inferior to the experimental results obtained from the complete network. This indicates that both the global information and local information are beneficial for our segmentation and recognition task from different perspectives. Therefore, when removing both the global and local modules, the model performance becomes extremely poor. The purpose of the fine-tuning module is to make the self supervised pre-trained model better adapt to the specific downstream task. Hence, when the fine-tuning module is removed, the model does not adapt well to the recognition and segmentation of mixed-type WBM defect patterns. To sum up, each module is proven to be crucial for the performance of the model through the ablation experiments.

## 5. Conclusions

In this paper, we introduce a self-supervised contrastive learning model designed for the classification and segmentation of mixed-type WBM defect patterns. The experimental results indicate that our model surpasses other self-supervised contrastive learning approaches. Furthermore, we demonstrate that the performance of our proposed model does not heavily depend on labeled data, but more self-supervised unlabeled pre-training data can improve the model’s performance. And the ablation study further assesses the contribution of each model component to the overall performance. In practical scenarios where large amounts of unlabeled WBM data can be easily obtained, our proposed model will have significant real-world applications. However, in the process of chip manufacturing, even minor defects can lead to chip failure or performance degradation. Therefore, the requirements for defect detection are extremely stringent. We will focus on improving the detection performance with a limited number of labeled samples. In future research, we aim to integrate network architecture optimizations with self-supervised learning to increase model evaluation accuracy, reduce processing time, and lower computational resource demands. We also plan to extend the application of our model to other fields, such as medical image segmentation and remote sensing image analysis.

## Figures and Tables

**Table 1:** Description of mixed-type WBM defect patterns.

Single-Type	C (C1)	D (C2)	EL (C3)	ER (C4)
	L (C5)	S (C6)	NF (C7)	R (C8)
Two-Mixed-Type	C + EL (C9)	C + ER (C10)	C + L (C11)	C + S (C12)
	D + EL (C13)	D + ER (C14)	D + L (C15)	D + S (C16)
	EL + L (C17)	EL + S (C18)	ER + L (C19)	ER + S (C20)
	L + S (C21)	-	-	-
Three-Mixed-Type	C + EL + L (C22)	C + EL + S (C23)	C + ER + L (C24)	C + ER + S (C25)
	D + EL + L (C26)	D + EL + S (C27)	D + ER + L (C28)	D + ER + S (C29)
	C + L + S (C30)	D + L + S (C31)	EL + L + S (C32)	ER + L + S (C33)
Four-Mixed-Type	C + L + EL + S (C34)	C + L + ER + S (C35)	D + L + EL + S (C36)	D + L + ER + S (C37)

**Table 2:** Segmentation and classification results for normal and single-type defect patterns (%).

Category		C0	C1	C2	C3	C4	C5	C6	C7	C8
Segmentation	Pacc	-	98.69	96.40	95.95	98.02	96.90	91.49	98.74	90.11
	IoU	-	96.21	94.19	93.23	95.88	96.05	89.35	97.55	88.46
Classification	Accuracy	98.75	98.67	95.83	96.89	98.41	97.04	94.54	98.72	92.32
	Precision	96.66	97.24	93.46	91.89	98.74	97.30	92.07	95.41	88.74
	Recall	93.19	92.95	95.20	96.11	97.68	94.69	96.38	92.88	95.01

**Table 3:** Segmentation results for mixed-type defect patterns (%).

Category	C		D		EL		ER		L		S	
	Pacc	IoU	Pacc	IoU	Pacc	IoU	Pacc	IoU	Pacc	IoU	Pacc	IoU
C9	98.57	96.12	-	-	95.94	93.23	-	-	-	-	-	-
C10	98.66	96.19	-	-	-	-	97.94	95.48	-	-	-	-
C11	98.49	96.10	-	-	-	-	-	-	96.88	96.01	-	-
C12	98.48	96.10	-	-	-	-	-	-	-	-	91.48	89.35
C13	-	-	96.23	93.89	95.66	93.10	-	-	-	-	-	-
C14	-	-	96.31	94.08	-	-	97.91	95.47	-	-	-	-
C15	-	-	96.20	93.88	-	-	-	-	96.79	95.12	-	-
C16	-	-	96.22	93.85	-	-	-	-	-	-	91.46	89.34
C17	-	-	-	-	95.21	92.16	-	-	96.20	95.03	-	-
C18	-	-	-	-	95.29	92.17	-	-	-	-	91.34	89.19
C19	-	-	-	-	-	-	97.82	95.40	96.77	95.13	-	-
C20	-	-	-	-	-	-	97.77	95.35	-	-	91.22	89.15
C21	-	-	-	-	-	-	-	-	96.18	94.99	90.79	88.94
C22	98.43	96.02	-	-	95.32	92.20	-	-	96.14	94.90	-	-
C23	98.42	96.02	-	-	95.32	92.21	-	-	-	-	90.19	88.03
C24	98.45	96.04	-	-	-	-	97.52	95.06	96.02	94.78	-	-
C25	98.45	96.03	-	-	-	-	97.55	95.07	-	-	90.12	87.94
C26	-	-	95.18	91.98	93.40	91.03	-	-	94.99	92.11	-	-
C27	-	-	95.15	91.90	93.39	91.03	-	-	-	-	90.10	89.90
C28	-	-	95.20	91.92	-	-	96.18	94.89	95.03	92.12	-	-
C29	-	-	95.18	91.97	-	-	96.15	94.87	-	-	90.10	87.89
C30	98.39	95.95	-	-	-	-	-	-	94.95	92.03	90.02	87.86
C31	-	-	95.11	91.86	-	-	-	-	94.92	92.02	90.01	87.85
C32	-	-	-	-	93.08	90.02	-	-	94.86	91.95	89.90	87.80
C33	-	-	-	-	-	-	96.05	94.80	94.87	91.95	89.90	87.81
C34	97.98	95.07	-	-	92.11	89.20	-	-	93.87	90.26	88.40	85.98
C35	98.00	95.08	-	-	-	-	95.11	93.57	93.89	90.27	88.41	85.98
C36	-	-	93.85	90.07	92.03	88.79	-	-	93.08	89.88	88.16	85.21
C37	-	-	93.90	90.10	-	-	94.79	93.14	93.11	89.95	88.18	85.25

**Table 4:** Classification results for mixed-type defect patterns (%).

Category	Accuracy	Precision	Recall
C9	95.62	98.67	93.30
C10	96.19	97.98	94.02
C11	94.45	97.56	93.88
C12	93.87	92.43	98.14
C13	91.92	93.00	97.44
C14	93.85	94.86	95.12
C15	91.68	96.33	92.88
C16	91.61	91.44	98.05
C17	94.31	97.87	93.30
C18	92.41	91.55	97.20
C19	92.84	92.34	94.99
C20	92.69	90.59	96.22
C21	91.88	96.81	91.74
C22	92.45	89.84	93.10
C23	89.97	95.44	92.13
C24	91.93	98.01	90.74
C25	90.76	93.71	89.67
C26	90.55	91.35	97.28
C27	89.68	91.55	96.98
C28	92.61	90.24	98.00
C29	89.77	99.01	92.74
C30	90.18	98.12	94.17
C31	89.11	94.38	96.05
C32	90.56	97.35	93.69
C33	90.34	92.44	96.77
C34	88.76	88.29	95.13
C35	89.52	91.30	93.27
C36	87.41	96.66	89.35
C37	88.49	87.80	94.06

**Table 5:** Comparison with other methods using limited labeled data on WBM defect pattern recognition and segmentation task (%).

Methods	Segmentation (Overall)		Recognition (Overall)		
	mPacc	mIoU	Accuracy	Precision	Recall
Jigsaw	83.66	76.19	80.94	78.44	81.71
SimCLR	83.43	75.85	80.41	75.96	78.13
MoCo v2	84.11	78.52	82.46	80.08	83.25
Ours	95.59	92.19	93.57	94.43	94.78

**Table 6:** Experimental results with different amounts of pre-training unlabeled data (%).

Different Amounts of Pre-Training Unlabeled Data		1%	25%	50%	75%	100%
		(152)	(3800)	(7600)	(11,400)	(15,200)
Segmentation results (Overall)	mPacc	31.12	40.55	72.64	89.33	95.59
	mIoU	29.33	40.17	63.81	82.30	92.19
Classification results (Overall)	Accuracy	27.94	38.55	70.66	86.45	93.57
	Precision	28.60	41.07	70.23	85.04	94.43
	Recall	30.41	45.02	71.38	88.10	94.78

**Table 7:** Experimental results with different amounts of fine-tuning labeled data (%).

Different Amounts of Fine-Tuning Labeled Data		1%	5%	10%	25%	50%	100%
		(152)	(760)	(1520)	(3800)	(7600)	(15,200)
Segmentation results (Overall)	mPacc	56.32	70.92	88.25	95.54	95.72	95.80
	mIoU	48.67	63.11	81.55	92.10	92.19	92.23
Classification results (Overall)	Accuracy	57.08	71.10	87.55	93.49	93.60	93.64
	Precision	58.68	75.43	89.02	94.38	94.92	95.17
	Recall	52.87	74.16	85.39	94.77	95.06	95.33

**Table 8:** Results of ablation experiments on exploring the effectiveness of each module (%).

Modules			Segmentation (Overall)		Classification (Overall)		
Global	Local	Fine-Tuning	mPacc	mIoU	Accuracy	Precision	Recall
√	√	√	95.59	92.19	93.57	94.43	94.78
√	√		75.09	71.54	72.64	75.16	71.81
√		√	84.11	78.52	82.46	80.08	83.25
	√	√	86.44	80.22	84.10	80.59	85.67
		√	52.76	45.28	50.49	49.77	55.01

## Data Availability

The datasets used in this study are available from the corresponding author upon reasonable request. These data were used under license for the current study and cannot be redistributed without permission from the data provider.
